# Genetic variations in anti-diabetic drug targets and COPD risk: evidence from mendelian randomization

**DOI:** 10.1186/s12890-024-02959-1

**Published:** 2024-05-15

**Authors:** Yue Su, Youqian Zhang, Jinfu Xu

**Affiliations:** 1 Department of Respiratory and Critical Care Medicine, Shanghai Pulmonary Hospital, School of Medicine, Tongji University, No. 507 Zhengmin Road, Shanghai, 200433 China; 2 Yangtze University, Jingzhou, Hubei Province 434000 China

**Keywords:** Mendelian randomization, Antidiabetic drugs, Chronic obstructive lung disease, Lung function

## Abstract

**Background:**

Previous research has emphasized the potential benefits of anti-diabetic medications in inhibiting the exacerbation of Chronic Obstructive Pulmonary Disease (COPD), yet the role of anti-diabetic drugs on COPD risk remains uncertain.

**Methods:**

This study employed a Mendelian randomization (MR) approach to evaluate the causal association of genetic variations related to six classes of anti-diabetic drug targets with COPD. The primary outcome for COPD was obtained from the Global Biobank Meta-analysis Initiative (GBMI) consortium, encompassing a meta-analysis of 12 cohorts with 81,568 cases and 1,310,798 controls. Summary-level data for HbA1c was derived from the UK Biobank, involving 344,182 individuals. Positive control analysis was conducted for Type 2 Diabetes Mellitus (T2DM) to validate the choice of instrumental variables. The study applied Summary-data-based MR (SMR) and two-sample MR for effect estimation and further adopted colocalization analysis to verify evidence of genetic variations.

**Results:**

SMR analysis revealed that elevated KCNJ11 gene expression levels in blood correlated with reduced COPD risk (OR = 0.87, 95% CI = 0.79–0.95; *p* = 0.002), whereas an increase in DPP4 expression corresponded with an increased COPD incidence (OR = 1.18, 95% CI = 1.03–1.35; *p* = 0.022). Additionally, the primary method within MR analysis demonstrated a positive correlation between PPARG-mediated HbA1c and both FEV1 (OR = 1.07, 95% CI = 1.02–1.13; *P* = 0.013) and FEV1/FVC (OR = 1.08, 95% CI = 1.01–1.14; *P* = 0.007), and a negative association between SLC5A2-mediated HbA1c and FEV1/FVC (OR = 0.86, 95% CI = 0.74–1.00; *P* = 0.045). No colocalization evidence with outcome phenotypes was detected (all PP.H4 < 0.7).

**Conclusion:**

This study provides suggestive evidence for anti-diabetic medications' role in improving COPD and lung function. Further updated MR analyses are warranted in the future, following the acquisition of more extensive and comprehensive data, to validate our results.

**Supplementary Information:**

The online version contains supplementary material available at 10.1186/s12890-024-02959-1.

## Introduction

Chronic obstructive pulmonary disease (COPD), is a progressive lung disease characterized by poorly reversible airflow blockage and persistent lung inflammation [1], contributing to increased resistance to airflow in the small conducting airways, reduced compliance of the lungs, and progressive airflow obstruction and air trapping [2]. Owing to the lung is a complex and vulnerable organ that is exposed to smoking, environmental degradation, and occupational hazards, the prevalence rate of COPD is increasing [3]. Based on the systematic analysis for the Global Burden Disease Study 2019, lower respiratory infection was the 3rd and COPD was the 6th cause of death, and the incidence rates vary by country and region, with higher rates in low- and middle-income countries and among people who smoke or work in industries with exposure to lung irritants [4]. The treatment for COPD includes medications, oxygen therapy, pulmonary rehabilitation, surgery, and lifestyle changes.

Diabetes mellitus is a chronic and metabolic disease, involving inappropriately increased blood glucose levels and systematic inflammatory responses accompanied by decreased insulin synthesis, insulin resistance (IR) or reduced metabolic response to insulin in many tissues [5]. To date, the major classes of oral antihyperglycemic medications include biguanides (e.g., metformin), thiazolidinedione (TZD), sulfonylureas, glucagon-like peptide 1 (GLP-1) receptor agonists, Insulin/insulin analogues (i.e., INSR), sodium-glucose cotransporter (SGLT2) inhibitors, dipeptidyl peptidase-IV (DPP-IV) inhibitors, and α-glucosidase inhibitors. Several studies have demonstrated the beneficial effect of antidiabetic drugs on COPD and its exacerbations. Pradhan et al. have demonstrated that GLP-1 receptor agonists and SGLT-2 inhibitors were capable of reducing severe exacerbations compared to sulfonylureas in patients diagnosed with COPD. However, the use of DPP-4 inhibitors did not clearly show a reduced risk of exacerbations in COPD [6]. Similarly, Au *et al* have suggested that SGLT2 inhibitor was associated with a decreased risk of incidence and exacerbations of obstructive airway diseases (OAD) compared with DPP4 inhibitor [7]. However, Hitchings et al. have found that metformin did not effectively decrease blood glucose levels in non-diabetic patients admitted to the hospital due to exacerbations of COPD, and no beneficial effect on CRP downregulation or clinical outcomes was identified [8]. However, to date, the role of antidiabetic drugs on COPD risk is inconclusive, and the confounding bias and conflicting results contribute to uncertain causation between antihyperglycemic medications and COPD.

Mendelian randomization is a statistical technique used in epidemiology and genetics to investigate causal relationships between an exposure (or risk factor) and an outcome, utilizing genetic variants or alleles as instrumental variables to estimate the causal effect of an exposure on an outcome. Importantly, MR is able to provide evidence for causality by leveraging genetic variants as instrumental variables, which helps overcome issues such as reverse causality and confounding over traditional observational studies and randomized controlled trials over traditional observational studies and randomized controlled trials. It is also less prone to biases introduced by self-reporting or recall bias. Hence, we performed a two-sample MR analysis in this study to explore the association of antidiabetic drugs with COPD.

## Materials and methods

### Study design

**Fig. 1:**
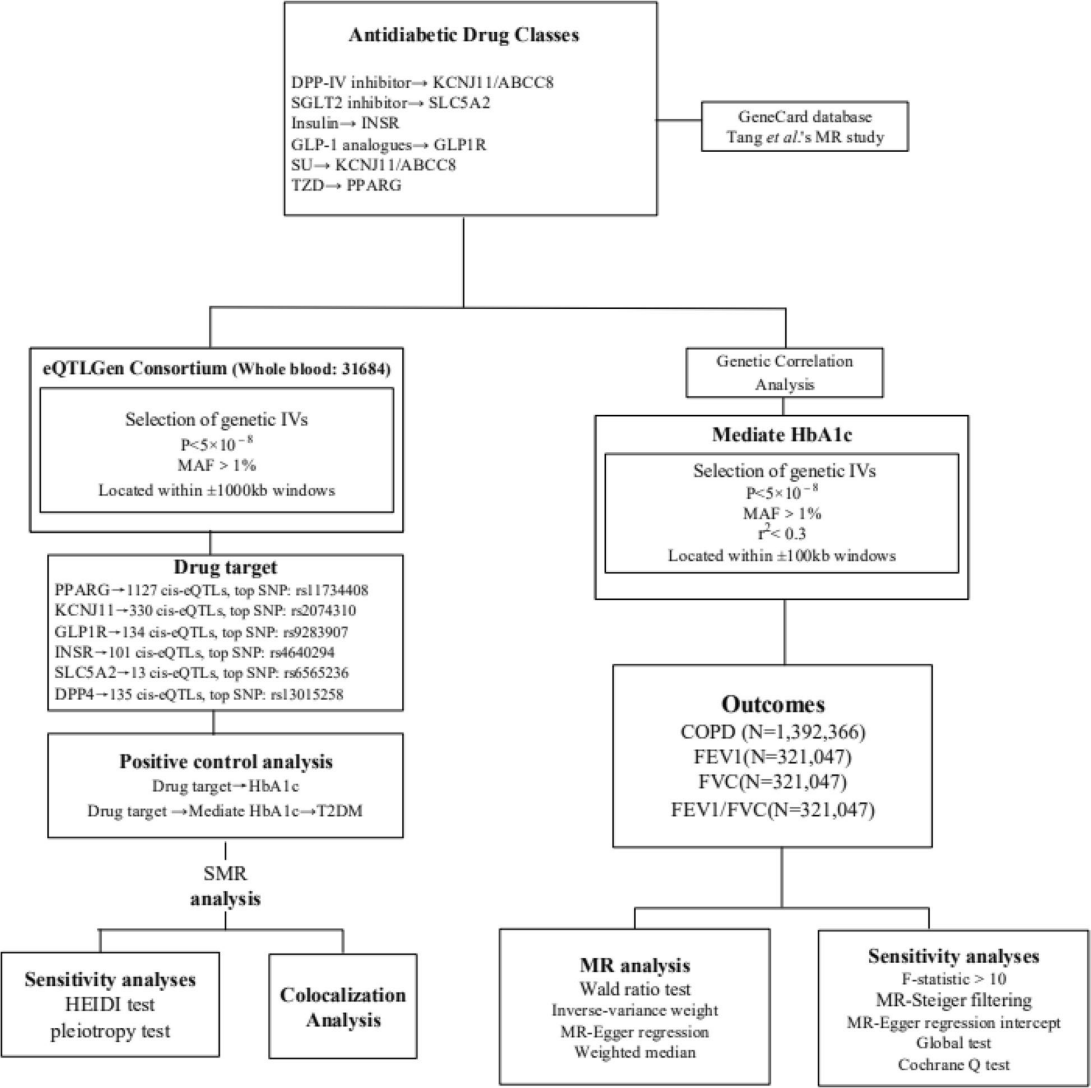
Flowchart of the study analysis

The foundational data for this study were procured from publicly accessible, summary-level datasets originating from Genome-Wide Association Studies (GWAS) and Expression Quantitative Trait Loci (eQTL) studies. A description of summary statistics data sources is available in Supplementary file 1: Table S1 and Supplementary file 2. Figure 1 displays the details of the Mendelian Randomization (MR) design. All included GWAS studies have received approval from the relevant institutional review boards. As this research involves secondary analysis of publicly available data, no additional ethical approval was required.

### Selection of genetic instrumental variables

In this study, the GeneCard database (https://www.genecards.org/) and drug targets used in this MR study [9], we identified the final drug targets analysed, namely TZD (i.e. PPARG), SU (i.e. KCNJ11 and ABCC8), GLP-1 analogues (i.e. GLP1R), Insulin/insulin analogues (i.e. INSR), SGLT2 inhibitor (i.e. SLC5A2), DPP-IV inhibitor (i.e. DPP4), metformin (i.e. PRKAB1, ETFDH, *etc*.). Owing to the controversy surrounding metformin's protein target and the largely unknown molecular basis of its physiological effects [10], we excluded metformin from further analytical consideration.

Summary-level data for eQTLs were sourced from the eQTLGen Consortium (https://www.eqtlgen.org/). We pinpointed common eQTLs single-nucleotide polymorphisms (SNPs) with a minor allele frequency (MAF) over 1% [11] that demonstrated a statistically significant correlation (*p* < 5.0 × 10^−8^) with PPARG, KCNJ11, GLP1R, INSR, SLC5A2 and DPP4 expression in blood. ABCC8 lacked significant eQTL levels and was therefore excluded. To generate genetic tools in this study, only cis eQTLs were included. These were defined as eQTLs situated within a 1 Mb range on either side of the encoding gene.

SNPs associated with HbA1c levels at genome-wide significance levels (*p* < 5.0 × 10^−8^) were selected from the target genes (± 100 kb of gene location) for each drug, based on the methodology used in the previous study [12]. HbA1c was obtained from UKB. To maximize the strength of the instrument for each drug, SNPs used as instruments were allowed to be in low weak linkage disequilibrium (r2 < 0.30) with each other. Finally, four drug targets were included in the study (PPARG, KCNJ11, GLP1R and SLC5A2). Additionally, we employed F-statistics (F = beta²/se²; beta for the SNP-exposure association; variance (se)) to examine the presence of weak instrumental variables [13]. The higher the F-statistic, the stronger the instrumental strength indicated. It was a requirement that all included SNPs have an F-statistic greater than 10. In MR analyses, MR-Steiger filtering was utilized to enhance the robustness of our findings by excluding variants manifesting stronger associations with the outcomes than with the exposure [13].

### Outcome

The primary outcome was COPD, derived from the database with the largest sample size of GWAS data available, the Global Biobank Meta-analysis Initiative (GBMI).

The GBMI [14], covering GWAS meta-analyses of 12 biobanks (BioMe, BioVU, Colorado Center for Personalised Medicine, Estonian Biobank, FinnGen, Generation Scotland, HUNT, Lifelines, Massachusetts General Brigham Biobank FinnGen, Generation Scotland, HUNT, Lifelines, Massachusetts General Brigham Biobank, Michigan Genomics Initiative, UCLA Precision Health Biobank, UK Biobank), ultimately including a European population of 81568 cases and 1310798 controls.

For secondary outcomes, lung function traits were examined, specifically Forced Expiratory Volume in 1-second (FEV1), Forced Vital Capacity (FVC), and the FEV1/FVC ratio. We utilized data from the most extensive presently available lung function GWAS by Shrine et al., which reported 279 genome-wide significant SNPs (*p* < 5×10^−9^) from a population of 321,407 of European ancestry [15]. The study adjusted for age, age^2^, height, and smoking status.

### Statistical analyses

#### Primary MR analysis

We employed the Summary-data-based Mendelian Randomization (SMR) approach to generate effect estimations using eQTLs as instrumental variables. This method enables a comprehensive exploration of the association between a specific gene's expression level and the desired outcome, utilizing summary-level data from both GWAS and eQTL studies. We used SMR software, version 1.03 (https://cnsgenomics.com/software/smr/#Overview), for allele harmonization and subsequent analytical procedures. The Wald ratio test was applied to individual instrumental variables (IVs), followed by the multiplicative random-effects inverse-variance-weighted (IVW) method for estimating causal associations of multiple IVs (≥ 2), supplemented by the MR-Egger and weighted median methods. The IVW weighting is directly proportional to the Wald ratio estimate for each SNP and inversely proportional to the variance estimate of the Wald ratio for each SNP. IVW provides reliable and efficient estimates when all genetic variations are considered valid. The weighted median method performs better when at least half of the genetic variations are considered invalid, whereas the MR-Egger method is applied when all genetic variations are considered invalid.

### Genetic correlation analysis

Linkage Disequilibrium Score (LDSC) regression, applicable to summary-level GWAS data, serves as an effective approach to genetic correlation analysis of complex diseases or traits. It distinguishes true polygenic signals from confounding biases such as cryptic relatedness and population stratification. If the genetic correlation demonstrates both statistical and quantitative significance, the overall phenotype correlation cannot be entirely attributed to environmental confounding factors [16]. The LDSC tool (https://github.com/bulik/ldsc) assists us in evaluating the genetic correlations among HbA1c, COPD, FEV1, FVC, and the FEV1/FVC ratio.

### Colocalization analysis

To confirm the shared causal genetic variation of eQTL implicated in the outcome phenotype as identified by MR, we conducted a colocalization analysis using the R package Coloc (version 3.2-1) [17]. The genetic variant showing the strongest association with the exposure in the MR assessment, indicated by the lowest *P*-value, was selected as the reference variant. Genetic variants within a range of ± 100 kb of this reference variant was included in the study. The LD reference panel was based on the 1000 Genomes v3 European ancestry dataset. The criterion for colocalization was a posterior probability exceeding 0.7 for a shared causal variant (posterior probability of hypothesis 4 > 0.7).

### Sensitivity analysis

We initially conducted a positive control analysis to verify the validity of the two selected genetic instruments. As HbA1c reflects the average blood sugar level over the past 2–3 months due to long-term exposure of haemoglobin to blood sugar, we investigated the association of related exposures with HbA1c levels as a positive control study for eQTL tools. For the HbA1c GWAS tool, given that diabetes is the primary indication for hypoglycemic drugs, we conducted a positive control study by investigating the association of related exposures with T2DM.

Within the SMR analysis, we acknowledged that observed associations between gene expression and the outcome could result from a linkage scenario. To validate this, we utilized the heterogeneity in dependent instruments (HEIDI) test. A *P*-value less than 0.01 would indicate the possibility of an association due to linkage [18]. Considering potential horizontal pleiotropy, we initially identified other genes significantly associated with genetic IVs (5×10^−8^) in close proximity to the top eQTL (within a 1 Mb window). We then performed SMR analysis to determine whether a single SNP was associated with the expression of multiple genes.

During the MR analysis, numerous tests were employed to ensure rigour and validity. Cochran's Q test was used to assess heterogeneity amongst the selected genetic variants, with a *P*-value of less than 0.05 indicating significant dissimilarity among the SNPs under investigation [16]. We further scrutinized any potential directional pleiotropy within our MR study by using MR-Egger regression [19]. Significant directional pleiotropy is indicated by a *P*-value less than 0.05 for the MR-Egger's intercept, despite this method's relative lack of precision [20]. We also employed the MR-PRESSO method to identify potential outliers and explore horizontal pleiotropy, inferred if the global *P*-value is under 0.05 [21]. This process allowed for the removal of outliers, thereby improving the accuracy of our correction. Following the Bonferroni adjustment for multiple testing, we regarded a *P*-value smaller than 0.0125 (derived from 0.05/4) as evidence of a statistically significant causal association. A *P*-value lower than 0.05 is considered to offer suggestive evidence of a possible causal relationship.

## Results

### Genetic instruments selection and genetic correlation between phenotypes

We identified 1127, 330, 134, and 13 cis-eQTLs for the drug target genes PPARG, KCNJ11, GLP1R, and SLC5A2, respectively, from the eQTLGen Consortium. The most significant cis-eQTL SNP for each drug target gene was selected as a genetic tool. Furthermore, from the GWAS summary data for HbA1c levels, we identified 23, 3, 3, and 4 SNPs within or near the genes PPARG, KCNJ11, GLP1R, and SLC5A2, respectively (Supplementary file 1: Table S2). The average F-statistic values of all instrument variations are 63.73, 81.69, 51.69, and 32.44, respectively, indicating robustness against weak instrument bias (Supplementary file 1: Table S3). In the positive control study, significant associations were observed between exposure to each drug and HbA1c when using the IVs represented by eQTLs (Supplementary file 1: Table S4). Similarly, significant associations were found between exposure to each medication and T2DM when using the IVs proposed by HbA1c GWAS, further confirming the effectiveness of the selected genetic instruments (Supplementary file 1: Table S5).

LDSC analysis unveiled robust genetic correlations between HbA1c and COPD (genetic correlation = 0.2048, *P* = 3.52×10^−13^), FEV1 (genetic correlation = -0.1179, *P* = 1.27×10^−7^), and FVC (genetic correlation = -0.1342, *P* = 2.88×10^−9^). A less pronounced genetic correlation was observed between HbA1c and FEV1/FVC (genetic correlation = 0.0221, *P* = 0.32). SNP-based liability-scale heritability h² for HbA1c, COPD, FEV1, FVC, and FEV1/FVC were 0.173, 0.011, 0.196, 0.191, and 0.189, respectively (Supplementary file 1: Table S6).

### Primary analysis

**Fig. 2:**
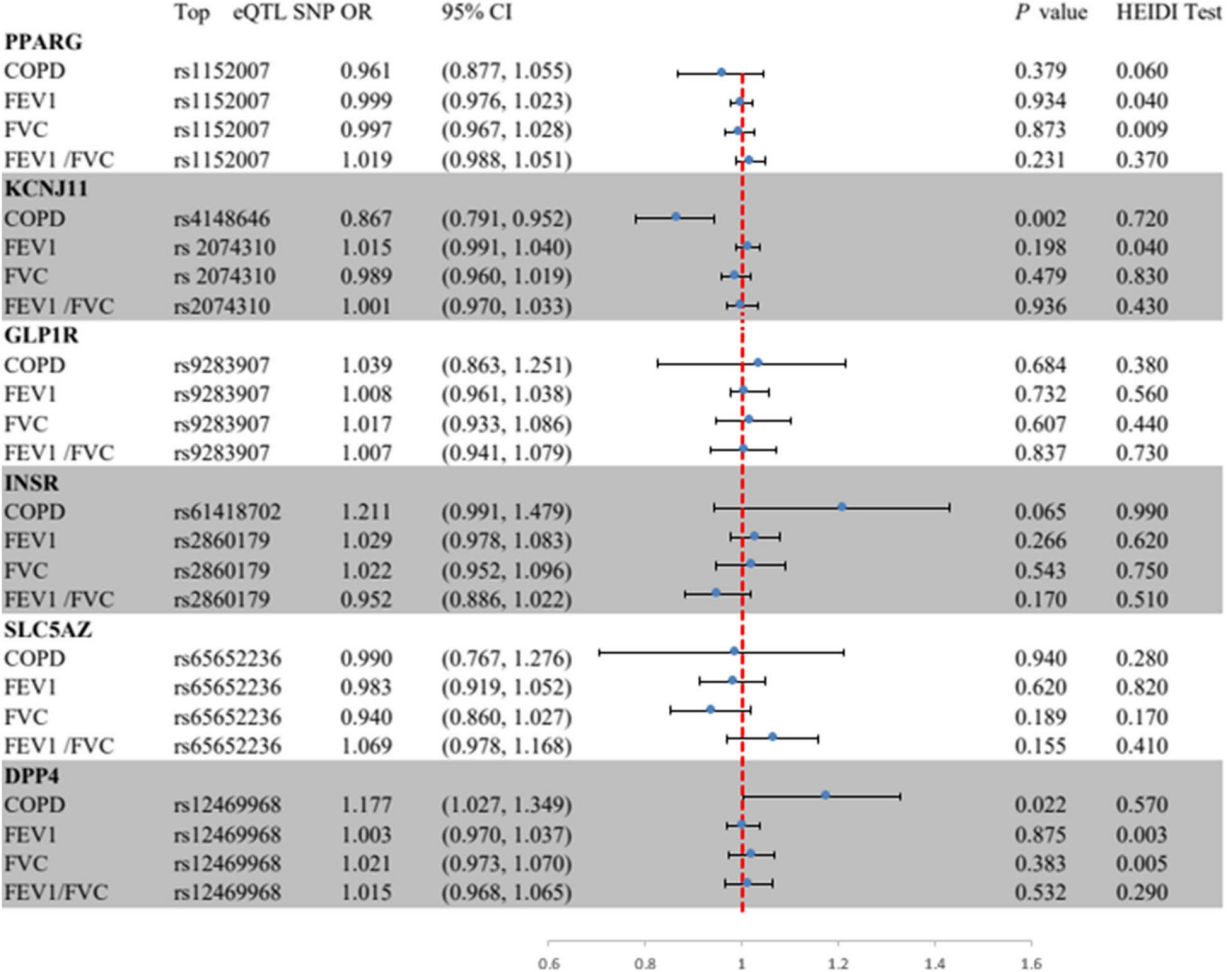
Summary-data-based Mendelian randomization (SMR) association between expression of gene PPARG, KCNJ11, GLP lR, INSR, SLC5A2, or DPP4 and outcome phenotypes. SMR method was used to assess the association. COPD, chronic obstructive pulmonary disease; FEV 1, forced expiratory volume in 1-second;FVC, forced vital capacity; HEIDI, heterogeneity in dependent instruments.

In Fig. 2, the SMR analysis provided suggestive evidence that a one standard deviation (SD) increases in the blood expression of the KCNJ11 gene reduced the incidence of COPD by 13% (OR = 0.87, 95% CI = 0.79–0.95; *P* = 0.002). Additionally, a one SD increase in the expression of the DPP4 gene in the blood increased the incidence of COPD by 18% (OR = 1.18, 95% CI = 1.03–1.35; *P* = 0.022), implying that HMGCR agonists and DPP4 inhibitors may reduce the risk of COPD. No significant association was found between the expression of PPARG, GLP1R, INSR, and SLC5A2 and outcome phenotypes (Supplementary file 1: Table S7).

**Fig. 3:**
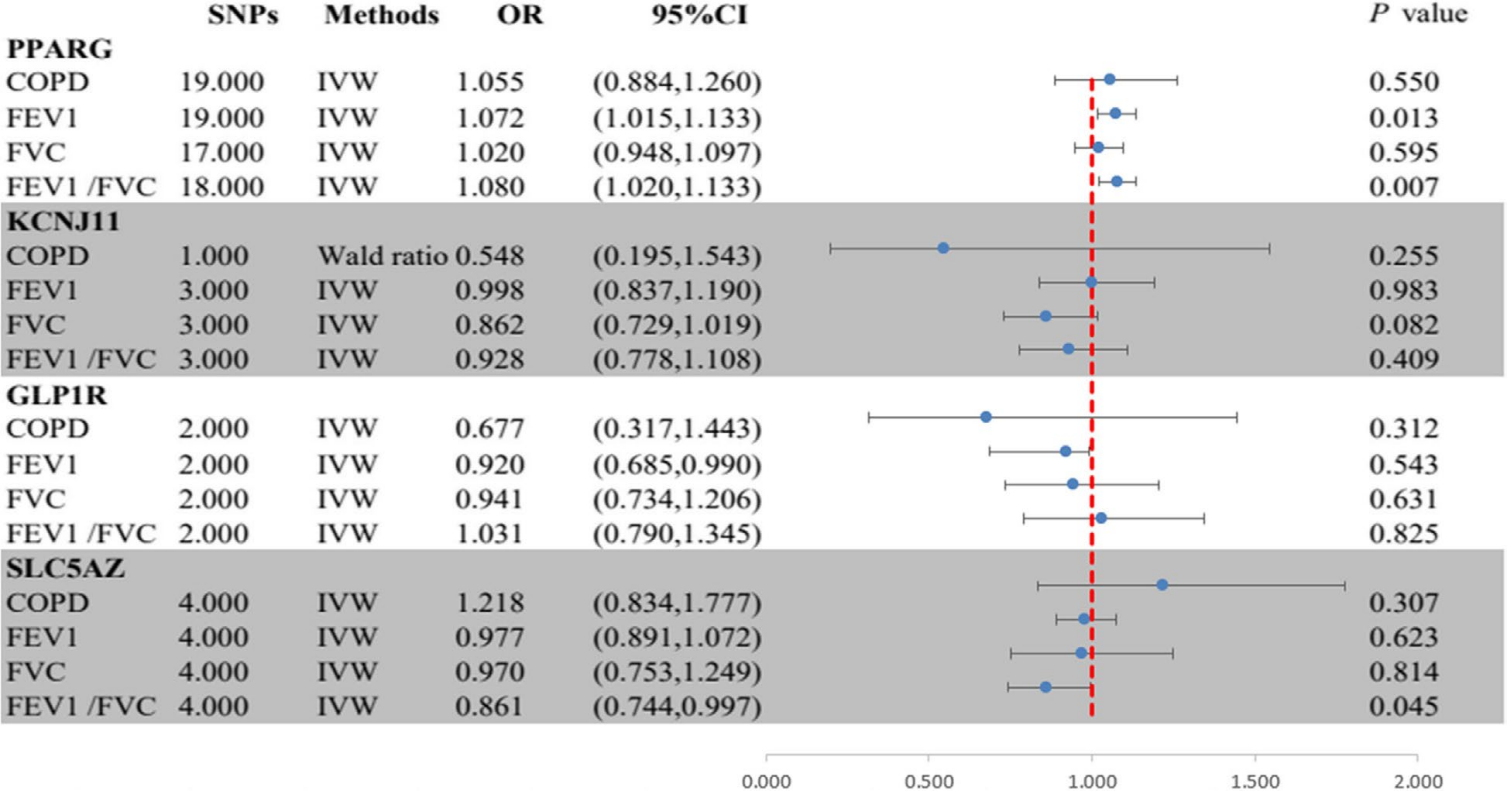
Summary results of the primary MR analyses between HbA1c mediated by gene PPARG, KCNJ11, GLP lR, or SLC5A2 and outcome phenotypes. IVW, Inverse-variance-weight; MR, Mendelian randomization; COPD, chronic obstructive pulmonary disease; FEV 1, forced expiratory volume in 1-second; FVC, forced vital capacity.

In Fig. 3, The MR analysis found strong evidence for an association between PPARG-mediated HbA1c and higher FEV1 (OR = 1.07, 95% CI = 1.02–1.13; *P* = 0.013) as well as FEV1/FVC (OR = 1.08, 95% CI = 1.01–1.14; *P* = 0.007), supporting a protective role of PPARG agonists on lung function (Fig. 2, Table S4). Suggestive evidence was observed for an association between SLC5A2-mediated HbA1c and lower FEV1/FVC (OR = 0.86, 95% CI = 0.74-1.00; *P* = 0.045), supporting a protective role of SLC5A2 inhibitors on lung function. No evidence was found of an association between HbA1c mediated by KCNJ11 or GLP1R and outcome phenotypes (Supplementary file 1: Table S8). All genes demonstrated no evidence of colocalization with the risk of outcome phenotypes (posterior probability of hypothesis 4 < 0.7) (Supplementary file 1: Table S9 and Supplementary file 3: Figure S1-24).

### Sensitivity analysis

The SMR analysis revealed that all observed associations were not due to linkage disequilibrium (HEIDI test *P* > 0.01), with the exception of associations between PPARG expression and FVC (*P* = 0.009), and DPP4 expression and FEV1 (*P* = 0.009) and FVC (*P* = 0.004) (Supplementary file 1: Table S10). We further explored potential horizontal pleiotropy between KCNJ11 and DPP4 expression and COPD and lung function, by examining associations between the expression of nearby genes significantly associated with the top eQTL SNPs of KCNJ11 and DPP4 and the outcomes. We identified 4 genes (including DPP4) and 8 genes (including KCNJ11) whose expressions were associated with instrumental variants (Table S). After screening, only two and eight genes, respectively, for DPP4 and KCNJ11, had available eQTLs at the genome-wide level (*P* < 5.0 × 10^−8^). The final results indicated that only KCNJ11 and DPP4 expression were significantly associated with COPD, ruling out the distortion of the results by horizontal pleiotropy (Supplementary file 1: Table S11).

In MR analysis, the Cochran Q test found no heterogeneity in the main reported results (all *P* > 0.05; Table S4), but unremovable heterogeneity was found in the PPARG-mediated HbA1c with FEV1 and FVC, and SLC5A2-mediated HbA1c with FVC. However, the intercepts in the MR-Egger regression and MR-PRESSO analyses indicated that the overall horizontal pleiotropy was not significant. Additionally, all instrumental variables passed MR-Steiger filtering, proving that the results remain robust (all *P* > 0.05; Supplementary file 1: Table S8).

## Discussion

The present study demonstrated the association between antidiabetic drugs and respiratory diseases through the SMR method. We found robust genetic correlations between HbA1c and COPD and lung function, including FEV1, and FVC. Moreover, with every SD increase in the expression of the KCNJ11 gene in the blood, the incidence of COPD is reduced by 13%, implying that KCNJ11 agonists may reduce the risk of COPD. Likewise, every SD increase in the expression of the DPP4 gene in the blood, the incidence of COPD increases by 18%. No significant association was found between the expression of PPARG, GLP1R, INSR, and SLC5A2 and outcome phenotypes.

PPARG, KCNJ11, GLP1R, and SLC5A2 are common therapeutic targets of antihyperglycemic drugs which are capable of inducing cellular differentiation, reducing cellular proliferation and inducing apoptosis [22]. PPARG is a member of the nuclear receptor superfamily of ligand-dependent transcription factors that is predominantly expressed in adipose tissue, modulating insulin sensitization and glucose metabolism [23]. Moreover, PPARG also reduces the morbidity in the experimental models of asthma, COPD and acute lung injury [24, 25]. Specifically, Solleti and colleagues have suggested that PPARG in the airway epithelial cell is able to modulate cigarette smoke-induced chemokine expression and emphysema susceptibility in mice [26]. Similarly, Karagiannis *et al* have found that PPARG is capable of controlling lipid uptake and transient storage in lipid droplets by influencing glucose levels [27]. GLP-1 as an incretin hormone possesses anti-inflammatory and immune-modulatory functions, and its receptor agonists contribute to decreased blood glucose levels [28]. Available studies have suggested that GLP-1 and its based agents have therapeutic potential in lung injury and COPD [29, 30]. Wei *et al* have shown that GLP-1-based pharmaceuticals presented reduced occurrence in most chronic lung diseases, except for interstitial lung disease [31]. Dogan *et al* have demonstrated that liraglutide use leads to increased FVC but no benefits in FEV1/FVC and 6-min walking distance [32]. Moreover, Rogliani and colleagues have shown that FEV1, FVC and maximal expiratory flow are significantly elevated after the GLP-1R treatment but not in the control group and insulin cohorts [33]. SLC5A2, namely the SGLT2 gene, was reported to be the drug target for SGLT2 inhibitors, hence the spatial distribution of SLC5A2 would provide insights into the target tissues of SGLT2 inhibitors [34]. Takashima and colleagues have demonstrated that low-dose luseogliflozin ameliorates ischemic brain injury in mice without glucose-lowering effects [35]. Similarly, Joki *et al* have shown that tofogliflozin modulates pulmonary vascular remodelling in mice with left heart disease [36]. Importantly, Jeong and co-workers have suggested that empagliflozin is able to improve respiratory function, reduce ischemia/reperfusion (I/R)-induced pulmonary edema and inflammatory cytokine production and protein concentration in the bronchoalveolar lavage (BAL) fluid through ERK1/2-mediated signalling pathway [37]. However, no significant association was found between the expression of PPARG, GLP-1R, and SLC5A2 and outcome phenotypes through SMR analysis, while KCNJ11 and DPP4 inhibitors may reduce the risk of COPD.

KCNJ11 encodes pancreatic β cell KATP channel pore-forming subunit (Kir6.2) with a key role in insulin secretion and glucose homeostasis, and mutation in KCNJ11 contributes to impaired blood glucose control [38]. A national cohort study showed that SU application was related to lower risks of cardiovascular events, ventilation use, pulmonary pneumonia, and mortality in the patients with COPD and diabetes [39]. Consistently, Wang *et al* have suggested that a duration-dependent beneficial impact of SU on severe COPD exacerbation was proved in patients with diabetes [40]. DPP4 encodes dipeptidyl peptidase 4 expressed on most cell types to decrease the expression of GLP-1 in the intestinal tract [41]. Kotnala and colleagues have shown that alveolar macrophages isolated from COPD patients presented higher DPP4 expression than that of healthy individuals, and DPP4 inhibition may alleviate the severity of haemophilus influenzae-induced COPD [42]. Similarly, the level of serum DPP4 was elevated in the patients with acute COPD exacerbation (AECOPD) [43]. However, a clinical study by Au *et al* found that DPP4 inhibitor use did not reduce the OAD exacerbations [7].

MR analysis is a genetic epidemiological approach that takes advantage of minimizing bias due to confounding and reverse causality and thus improves the causal inference [44]. In the present study, we found that no significant association was found between the expression of PPARG, GLP1R, INSR, and SLC5A2 and outcome phenotypes. Two-sample MR analyses have suggested that PPARG or SLC5A2-mediated HbA1c play an important role in lung function, suggesting a protective role of SLC5A2 inhibitors on lung function. Additionally, there was no evidence of an association between HbA1c mediated by KCNJ11 or GLP1R and outcome phenotypes. In the SMR analysis, only KCNJ11 and DPP4 expression were significantly associated with COPD. However, the intercepts in the MR-Egger regression and MR-PRESSO analyses indicated that the overall horizontal pleiotropy was not significant.

The primary advantage of this study lies in employing genetic tools to represent drug exposure, a strategy that reduces potential bias from external factors and prevents the issue of reverse causation. Furthermore, we utilized two distinct genetic instruments to mimic the drug under investigation. This approach adds credibility to the obtained effect estimates as they support each other. Additionally, we conducted several sensitivity analyses to assess the reliability of the genetic instruments and the assumptions made in this study. However, there are several limitations in this study. First, while we have conducted a range of sensitivity analyses to evaluate the assumptions of the MR study, it is important to acknowledge that the possibility of confounding bias and/or horizontal pleiotropy cannot be entirely ruled out. Second, it is important to note that the anticipated effects of drugs based on genetic predictions may not align with actual therapeutic outcomes. The genetic variants that serve as instruments for drug exposure are present from birth and lasts throughout one's lifetime. Consequently, our analyses cannot capture the impact of exposure to antidiabetic medications during specific periods of life. Third, it is worth noting that the eQTLs and GWAS data utilized in our research primarily stem from individuals with European ancestry, hence, extending these findings to other population groups is essential. Lastly, due to the use of summary-level data, we were unable to conduct subgroup analyses. Therefore, it is imperative to conduct further MR studies using individual-level data to obtain more comprehensive and detailed information to decide the individuals who are likely to be taking these drugs.

## Conclusion

To sum up, the MR analysis indicates a potential cause-and-effect connection between KCNJ11 and DPP4 inhibitors and COPD risk, recommending that clinical trials should be conducted to investigate whether antidiabetic drugs offer protective benefits against COPD. Additionally, more research is necessary to delve into the underlying mechanisms.

### Supplementary Information

**Supplementary Material 1.**

**Supplementary Material 2.**

**Supplementary Material 3.**

## Data Availability

The authors confirm that the data supporting the findings of this study within the article and its Supplementary material. Raw data analyzed for the present study are available from the corresponding author upon reasonable request.
