# Supplementary material for: Genetic variations in anti-diabetic drug targets and COPD risk: evidence from mendelian randomization
Source: BMC Pulm Med. 2024 May 15;24:240. doi: 10.1186/s12890-024-02959-1 (PMC11094874; doi:10.1186/s12890-024-02959-1)
Supplement: Supplementary file 2 — Supplementary Material 2. [file 12890_2024_2959_MOESM2_ESM.docx]

**Description of summary statistics data sources**

**eQTLGen Consortium (https://www.eqtlgen.org/phase1.html)**^1^

The eQTLGen Consortium is a collaborative organization consisting of multiple research institutions and laboratories across Europe, with the aim of exploring the genetic mechanisms of gene expression. The eQTLGen Consortium's research focuses primarily on gene expression in blood samples. They analyzed 31,684 blood and peripheral blood mononuclear cell (PBMC) samples from 37 datasets, which were pre-processed in a standardized way and analyzed by each cohort analyst. Of these samples, 25,482 (80.4%) were whole blood samples and 6,202 (19.6%) were PBMCs, and the majority of samples were of European ancestry. The gene expression levels of the samples were profiled using various techniques, including Illumina (N=17,421; 55%), Affymetrix U219 (N=2,767; 8.7%), and Affymetrix Hu-Ex v1.0 ST (N=5,075; 16%) expression arrays, as well as RNA-seq (N=6,422; 20.3%).

The eQTLGen Consortium's analysis revealed thousands of genetic loci and polygenic scores that regulate blood gene expression. They found that 88.2% of the genes expressed in blood showed a cis-eQTL effect, 32% showed a trans-eQTL effect, and 13% showed an eQTS effect. These results provide important genetic information on gene expression that can be used to prioritize genes associated with complex traits. In addition to these findings, the eQTLGen Consortium also discovered some important results. For example, they found distal trans-eQTLs that can affect gene expression over long distances, but these loci had low replication rates. To address this issue, the researchers used CHi-C technology and found that these loci were related to chromatin spatial structure, providing important clues for further research.

Furthermore, the eQTLGen Consortium studied the priority of intracellular trans-eQTLs and found that these loci can affect gene expression through multiple mechanisms, such as regulating transcription factor activity and affecting RNA splicing. Overall, the eQTLGen Consortium's research provides important insights into the genetic regulation of gene expression in blood samples and can be used to advance our understanding of complex traits.

**GWAS of chronic obstructive pulmonary disease (COPD) by GBMI Consortium**^2^**.**

**(**[**https://www.globalbiobankmeta.org/resources**](https://www.globalbiobankmeta.org/resources)**)**

The Global Biobank Meta-analysis Initiative (GBMI) Consortium is a collaborative network of 24 biobanks with over 2.2 million individuals, established to advance genetic discoveries for human diseases through the integration of genome-wide association studies (GWAS) from different biobanks worldwide. By pooling together data from multiple biobanks, the GBMI aims to increase the statistical power of GWAS and identify genetic variants associated with various human diseases. The GBMI has successfully integrated GWAS data from different biobanks and identified 317 known and 183 novel loci for 14 endpoints. The GBMI publicly releases summary statistics of biobank meta-analyses, promoting transparency and collaboration in the field of genetic research. Although there are differences in case definitions, recruitment strategies, and baseline characteristics, the integration of GWAS across different biobanks has been validated and has improved risk prediction, increased GWAS power for under-studied diseases, and nominated disease genes and candidate drugs through the integration of gene and protein expression data. This provides insights into the potential biology of human diseases and traits, making the GBMI Consortium a groundbreaking initiative with significant implications for the understanding and treatment of human diseases. COPD data source from the GBMI, covering GWAS meta-analyses of 12 biobanks (BioMe, BioVU, Colorado Center for Personalised Medicine, Estonian Biobank, FinnGen, Generation Scotland, HUNT, Lifelines, Massachusetts General Brigham Biobank FinnGen, Generation Scotland, HUNT, Lifelines, Massachusetts General Brigham Biobank, Michigan Genomics Initiative, UCLA Precision Health Biobank, UK Biobank), ultimately including a European population of 81568 cases and 1310798 controls. Because GBMI is a consolidated content, please extract the specific queue information from the corresponding original text.

**GWAS of lung function by Nick Shrine et al**^3^**.**

The analysis enhanced the quality control of spirometry, incorporating genome-wide association studies (GWAS) of forced expiratory volume in 1 second (FEV1), forced vital capacity (FVC), and the FEV1/FVC ratio in 321,047 individuals from the UK Biobank and 79,055 individuals from the SpiroMeta Consortium. Various statistical methods were applied to analyze the data. These analyses covered 19,819,130 autosomal variants present in both the UK Biobank and SpiroMeta. Additionally, peak expiratory flow (PEF) was assessed genome-wide in samples from both UK Biobank and SpiroMeta, involving up to 24,218 individuals. Quality control procedures were executed to ensure data accuracy and reliability, including filtering out samples with low-quality spirometry readings and removing genetic variants with low call rates or high levels of missing data.

The study design incorporating both two-stage and one-stage methods was adopted. Through this approach, 279 genetic markers associated with lung function were identified, 139 of which were newly discovered. The study also highlighted biological pathways and potential drug targets for COPD, as well as the pleiotropic effects of lung function-related variants. To determine whether the 279 signals were associated with other traits and diseases, a weighted genetic risk score (GRS) was calculated for up to 379,337 UK Biobank samples, and a phenome-wide association study (PheWAS) was performed, using the GRS as the exposure. The study covered UK Biobank baseline measures (from questionnaires and physical measures), self-reported medication usage, operative procedures, as well as those documented in Office of Population Censuses and Surveys codes from the electronic health record. Self-reported disease variables and those from hospital episode statistics (ICD-10 codes truncated to three-character codes and combined in block and chapter groups) were also included, combining these where possible to maximize power. The GRS analysis incorporated 2,453 traits, and the single-variant analysis comprised 2,411 traits. Analyses were conducted in unrelated individuals of European ancestry (with KING kinship coefficient <0.0442) and were adjusted for age, sex, genotyping array, and ten principal components. Logistic and linear models were fitted for binary and quantitative outcomes, respectively. False discovery rates were calculated according to the number of traits in the GRS and single-variant PheWAS (2,453 or 2,411, respectively).

**GWAS of Type 2 Diabetes Mellitus (T2DM) by Mahajan A et al**^4^ **.**

In this study, data from 898,130 individuals of European ancestry were utilized, with 9% being patients with T2DM. The sample size is ample to identify known T2DM-associated regions and furthermore, allowed the discovery of 135 new T2DM-associated areas. In addition, a power calculation was conducted, demonstrating our dataset to have >80% power to discern variants with a >5% allele frequency and 1.10 OR, or a 0.1% allele frequency with an OR of 1.60.

High-density genotypic data, derived from high-density imputation of a reference panel, were deployed. The reference panel, obtained through whole-genome sequencing of 15,220 Icelanders, contains 64,976 haplotypes. Quality control of genotypic data was undertaken using PLINK software, which included steps such as removing disqualified individuals (e.g., those with a missing rate exceeding 5%), discarding disqualified SNPs (e.g., SNPs with a missing rate over 5% or deviating from Hardy-Weinberg equilibrium), and excluding highly correlated individuals (e.g., those with an IBD > 0.2). Ultimately, genotypic data involving 2,543,887 SNPs were secured.

In the T2DM GWAS, phenotypic analysis of the sample was required. Phenotypic data from electronic medical records and questionnaires, including information on T2DM diagnosis, blood glucose levels, insulin levels, BMI, etc., were used. Quality control of phenotypic data, involving the elimination of outliers and missing values, was conducted. This yielded a phenotypic dataset encompassing T2DM patients and non-patients. Quality control is a crucial step, and various methods were employed for both genotypic and phenotypic data to ensure the reliability of our findings. In addition, matching of genotypic and phenotypic data was performed to confirm consistency.

In this paper, high-density genotypic data, derived from high-density imputation of a reference panel, was used. Phenotypic data, including information on T2DM diagnosis, blood glucose levels, insulin levels, BMI, etc., were collected from electronic medical records and questionnaires. Quality control was performed on both genotypic and phenotypic data to ensure the reliability of our findings. Ultimately, we obtained a genotypic dataset containing 2,543,887 SNPs and a phenotypic dataset containing both T2DM patients and non-patients. Through the analysis of these data, we discovered 135 new T2DM-associated regions and also identified 80 risk alleles with lower allele frequencies. Additionally, we identified 18 T2DM therapeutic targets with coding variations. These findings provide critical insights and directions for further genetic studies of T2DM.

**GWAS of Glycated haemoglobin (HbA1c) by** **UKB Consortium**^5^**.**

**(http://www.nealelab.is/uk-biobank)**

The GWAS study comprised a meta-analysis of six cohorts, with a total sample size of 419,434, including 226,723 women and 192,711 men. This is mainly composed of 1,502 Middle Easterners (0.4%), 5,290 Africans (1.3%), 922 Admixed Americans (0.2%), 8,329 Central and South Asians (2%), 2,566 East Asians (0.6%), and 400,825 Europeans (95.5%). The majority of participants in the study were over 50 years old on average. The National Glycohemoglobin Standardization Program (NGSP) percentage of HbA1c has been adjusted for age, sex, study-specific covariates, and genomic controls.

Data used in these analyses were derived from the UK Biobank, a large-scale open database that includes genotypic data from hundreds of thousands of individuals, matched with electronic health records and survey measures. From 2006 to 2010, the UK Biobank recruited 400,000 individuals aged between 69-2006 from around the country to participate in the project. They have undergone measures, provided blood, urine, and saliva samples for future analyses, and furnished detailed information about themselves, consenting to have their health status tracked. The fundamental aim of the study is to establish a resource to facilitate access to genetic association results (also known as summary statistics) for as many phenotypes as possible in as many different populations, particularly those underrepresented traditionally in previous genetic studies and largely excluded in most analyses using the widely-used UK Biobank resource.

Reference.

1. Võsa, U. *et al.* Large-scale cis- and trans-eQTL analyses identify thousands of genetic loci and polygenic scores that regulate blood gene expression. *Nat Genet* **53**, 1300–1310 (2021).

2. Zhou, W. *et al.* Global Biobank Meta-analysis Initiative: Powering genetic discovery across human disease. *Cell Genom* **2**, 100192 (2022).

3. Shrine, N. *et al.* New genetic signals for lung function highlight pathways and chronic obstructive pulmonary disease associations across multiple ancestries. *Nat Genet* **51**, 481–493 (2019).

4. Mahajan, A. *et al.* Fine-mapping type 2 diabetes loci to single-variant resolution using high-density imputation and islet-specific epigenome maps. *Nat Genet* **50**, 1505–1513 (2018).

5. Trait: Glycated haemoglobin - IEU OpenGWAS project. https://gwas.mrcieu.ac.uk/datasets/ukb-d-30750_irnt/.
