# Supplementary material for: Genetic variations in anti-diabetic drug targets and COPD risk: evidence from mendelian randomization
Source: BMC Pulm Med. 2024 May 15;24:240. doi: 10.1186/s12890-024-02959-1 (PMC11094874; doi:10.1186/s12890-024-02959-1)
Supplement: Supplementary file 3 — Supplementary Material 3. [file 12890_2024_2959_MOESM3_ESM.docx]

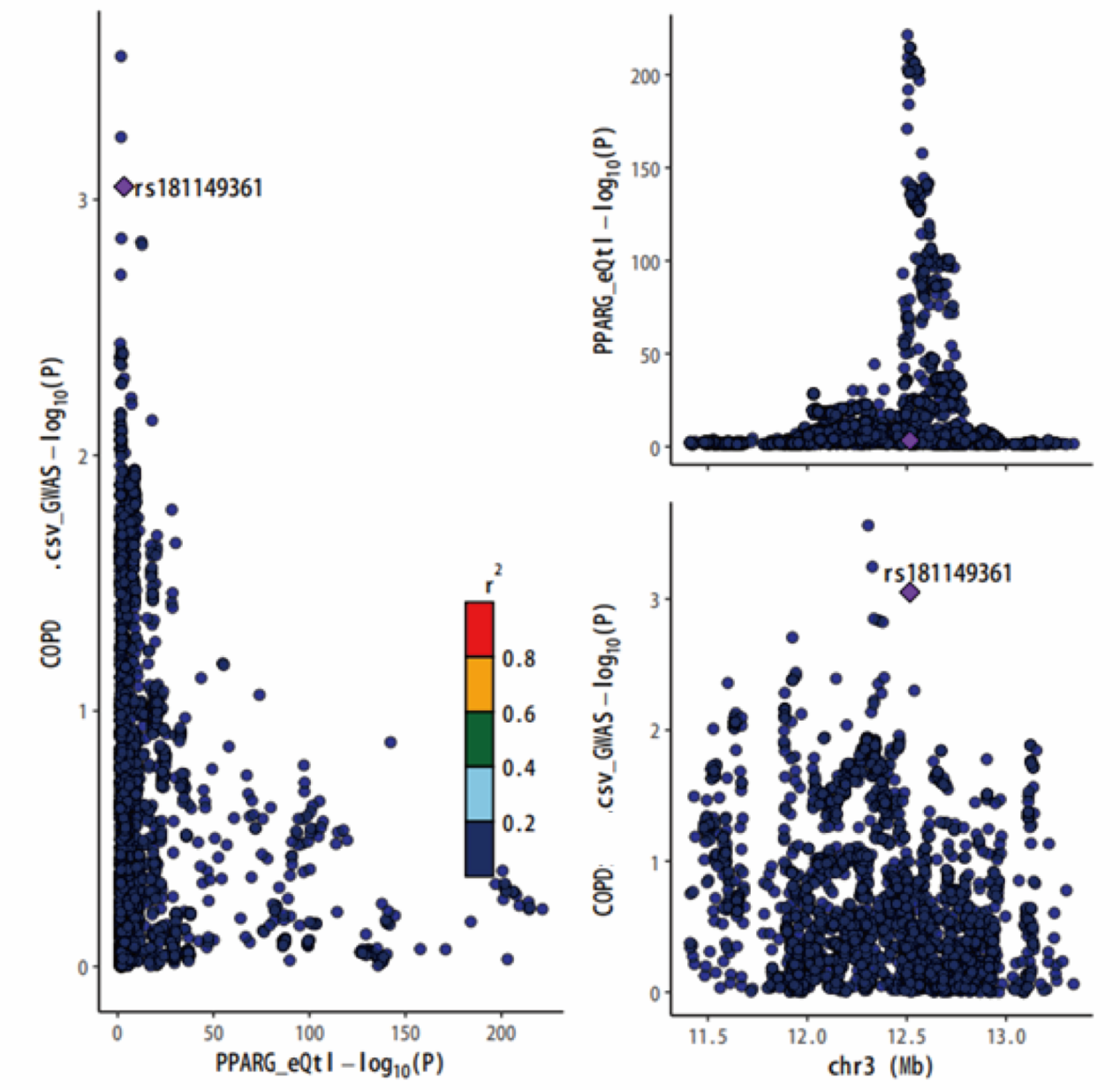


**Figure S1. Results of co-localization analysis of PPARG gene with chronic obstructive pulmonary disease (COPD).**

Each point represents a variant with chromosomal position on the x-axis (within 500-kb regions of each sentinel variant for candidate proteins) and the -log10(p-value) on the y-axis. Variants are coloured by linkage disequilibrium with the sentinel variant. Blue lines show the recombination rate; gene locations are shown at the bottom of the plot.


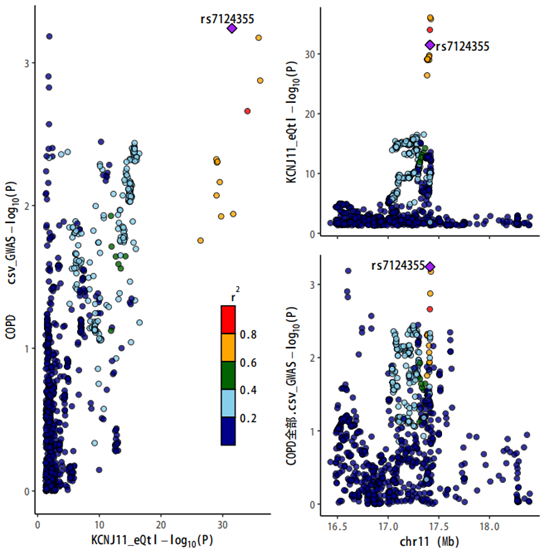


**Figure S2. Results of co-localization analysis of KCNJ11 gene with chronic obstructive pulmonary disease (COPD).**

Each point represents a variant with chromosomal position on the x-axis (within 500-kb regions of each sentinel variant for candidate proteins) and the -log10(p-value) on the y-axis. Variants are coloured by linkage disequilibrium with the sentinel variant. Blue lines show the recombination rate; gene locations are shown at the bottom of the plot.


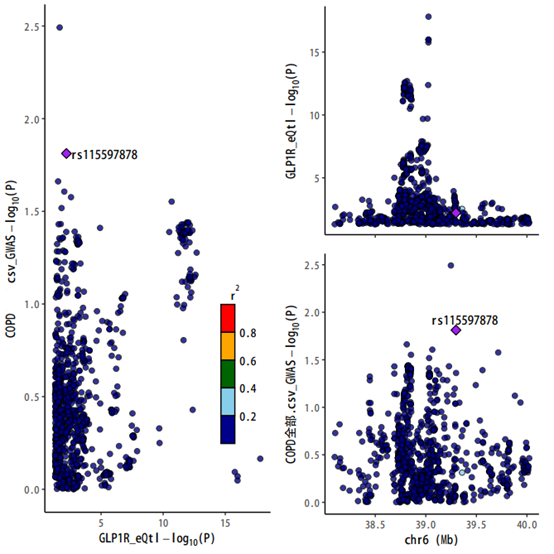


**Figure S3. Results of co-localization analysis of GLP1R gene with chronic obstructive pulmonary disease (COPD).**

Each point represents a variant with chromosomal position on the x-axis (within 500-kb regions of each sentinel variant for candidate proteins) and the -log10(p-value) on the y-axis. Variants are coloured by linkage disequilibrium with the sentinel variant. Blue lines show the recombination rate; gene locations are shown at the bottom of the plot.


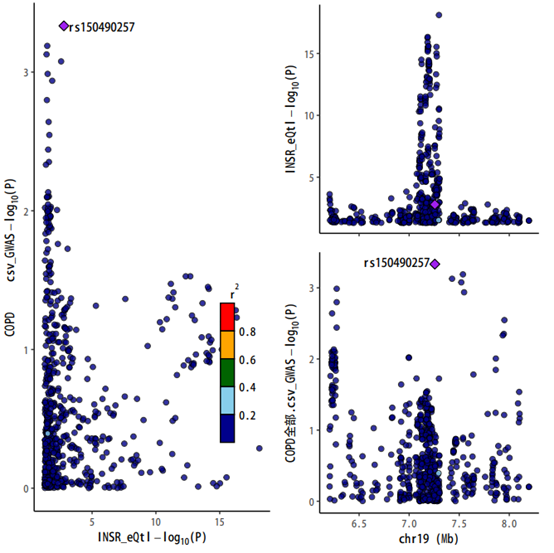


**Figure S4. Results of co-localization analysis of INSR gene with chronic obstructive pulmonary disease (COPD).**

Each point represents a variant with chromosomal position on the x-axis (within 500-kb regions of each sentinel variant for candidate proteins) and the -log10(p-value) on the y-axis. Variants are coloured by linkage disequilibrium with the sentinel variant. Blue lines show the recombination rate; gene locations are shown at the bottom of the plot.


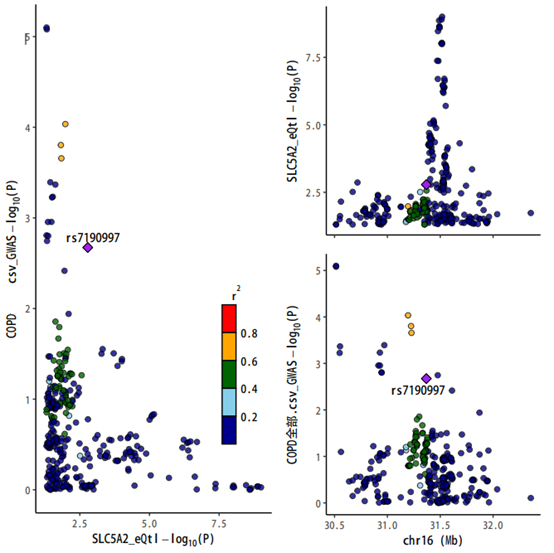


**Figure S5. Results of co-localization analysis of SLC5A2 gene with chronic obstructive pulmonary disease (COPD).**

Each point represents a variant with chromosomal position on the x-axis (within 500-kb regions of each sentinel variant for candidate proteins) and the -log10(p-value) on the y-axis. Variants are coloured by linkage disequilibrium with the sentinel variant. Blue lines show the recombination rate; gene locations are shown at the bottom of the plot.


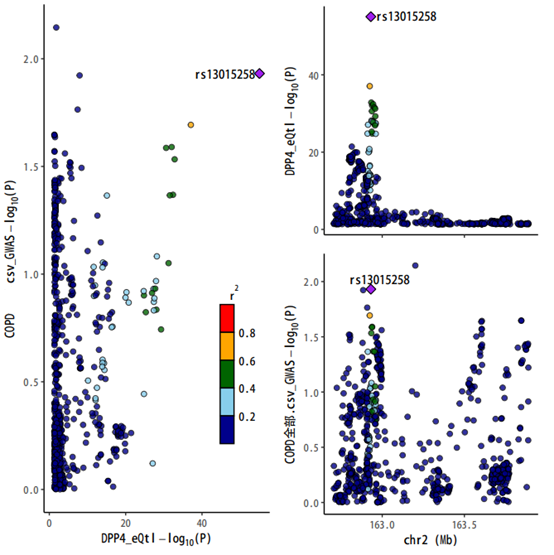


**Figure S6. Results of co-localization analysis of DPP4 gene with chronic obstructive pulmonary disease (COPD).**

Each point represents a variant with chromosomal position on the x-axis (within 500-kb regions of each sentinel variant for candidate proteins) and the -log10(p-value) on the y-axis. Linkage Variants colour variants by linkage disequilibrium with the sentinel variant. Blue lines show the recombination rate; gene locations are shown at the bottom of the plot.


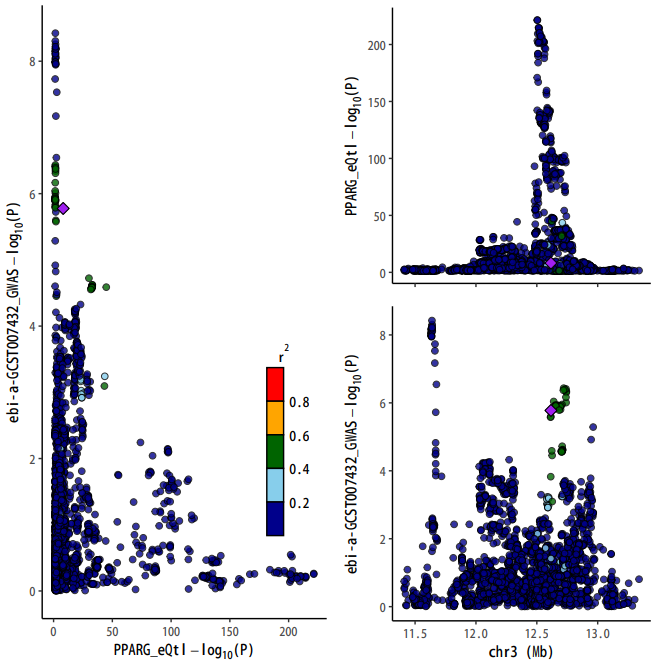


**Figure S7. Results of co-localization analysis of PPARG gene with Forced expiratory volume in 1-second (FEV1).**

Each point represents a variant with chromosomal position on the x-axis (within 500-kb regions of each sentinel variant for candidate proteins) and the -log10(p-value) on the y-axis. Variants are coloured by linkage disequilibrium with the sentinel variant. Blue lines show the recombination rate; gene locations are shown at the bottom of the plot.


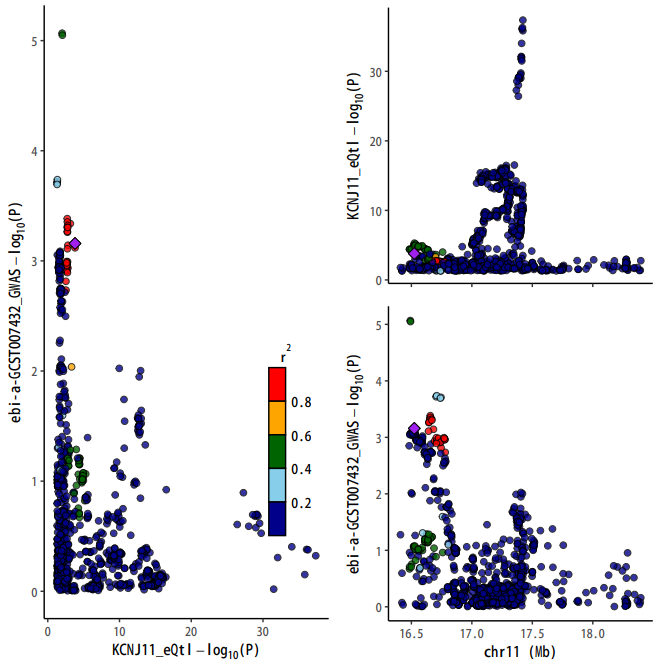


**Figure S8. Results of co-localization analysis of KCNJ11 gene with Forced expiratory volume in 1-second (FEV1).**

Each point represents a variant with chromosomal position on the x-axis (within 500-kb regions of each sentinel variant for candidate proteins) and the -log10(p-value) on the y-axis. Variants are coloured by linkage disequilibrium with the sentinel variant. Blue lines show the recombination rate; gene locations are shown at the bottom of the plot.


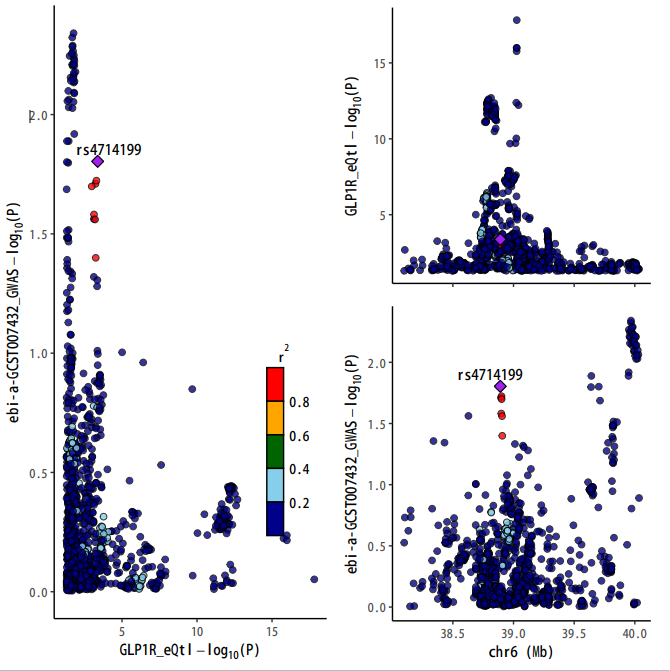


**Figure S9. Results of co-localization analysis of GLP1R gene with Forced expiratory volume in 1-second (FEV1).**

Each point represents a variant with chromosomal position on the x-axis (within 500-kb regions of each sentinel variant for candidate proteins) and the -log10(p-value) on the y-axis. Variants are coloured by linkage disequilibrium with the sentinel variant. Blue lines show the recombination rate; gene locations are shown at the bottom of the plot.


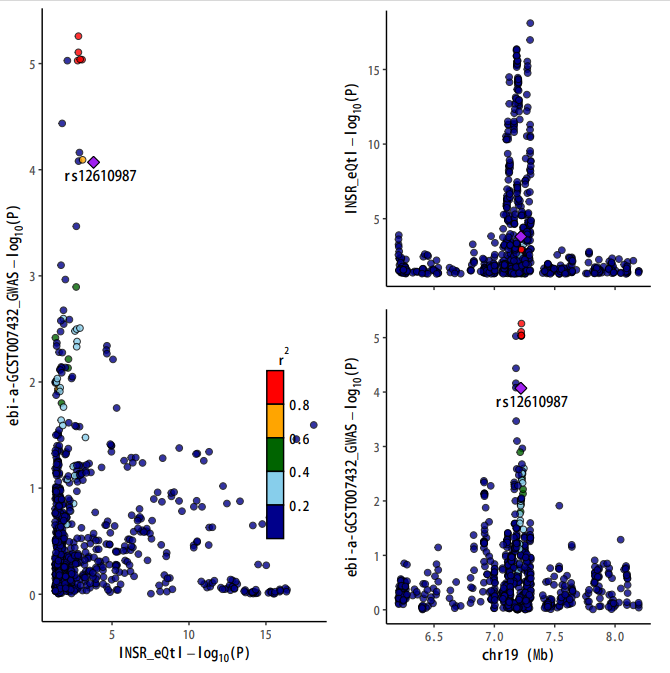


**Figure S10. Results of co-localization analysis of INSR gene with Forced expiratory volume in 1-second (FEV1).**

Each point represents a variant with chromosomal position on the x-axis (within 500-kb regions of each sentinel variant for candidate proteins) and the -log10(p-value) on the y-axis. Variants are coloured by linkage disequilibrium with the sentinel variant. Blue lines show the recombination rate; gene locations are shown at the bottom of the plot.


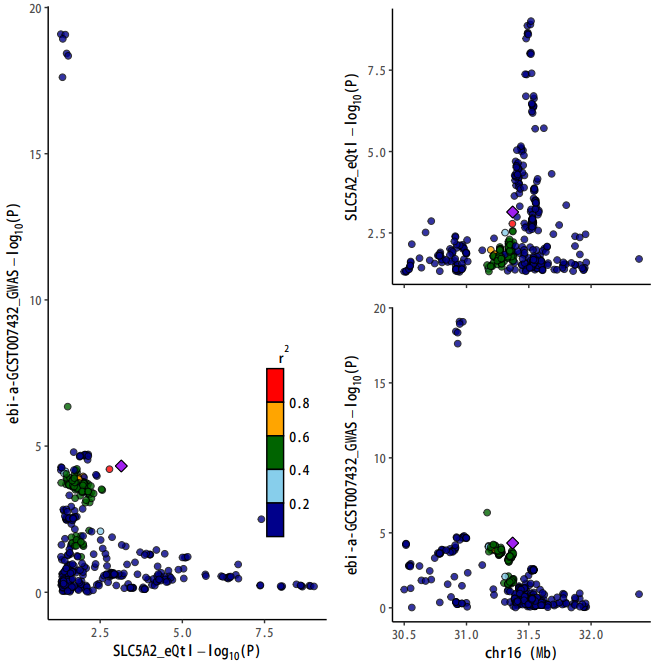


**Figure S11. Results of co-localization analysis of SLC5A2 gene with Forced expiratory volume in 1-second (FEV1).**

Each point represents a variant with chromosomal position on the x-axis (within 500-kb regions of each sentinel variant for candidate proteins) and the -log10(p-value) on the y-axis. Variants are coloured by linkage disequilibrium with the sentinel variant. Blue lines show the recombination rate; gene locations are shown at the bottom of the plot.


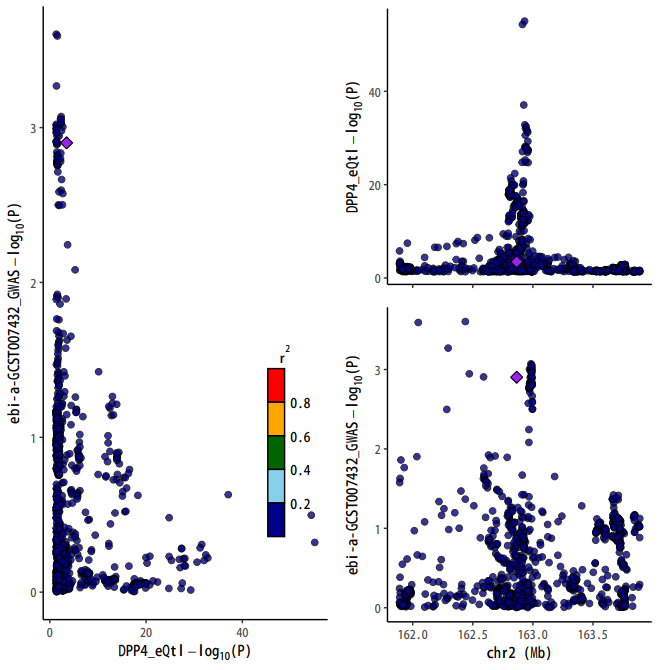


**Figure S12. Results of co-localization analysis of DPP4 gene with Forced expiratory volume in 1-second (FEV1).**

Each point represents a variant with chromosomal position on the x-axis (within 500-kb regions of each sentinel variant for candidate proteins) and the -log10(p-value) on the y-axis. Variants are coloured by linkage disequilibrium with the sentinel variant. Blue lines show the recombination rate; gene locations are shown at the bottom of the plot.


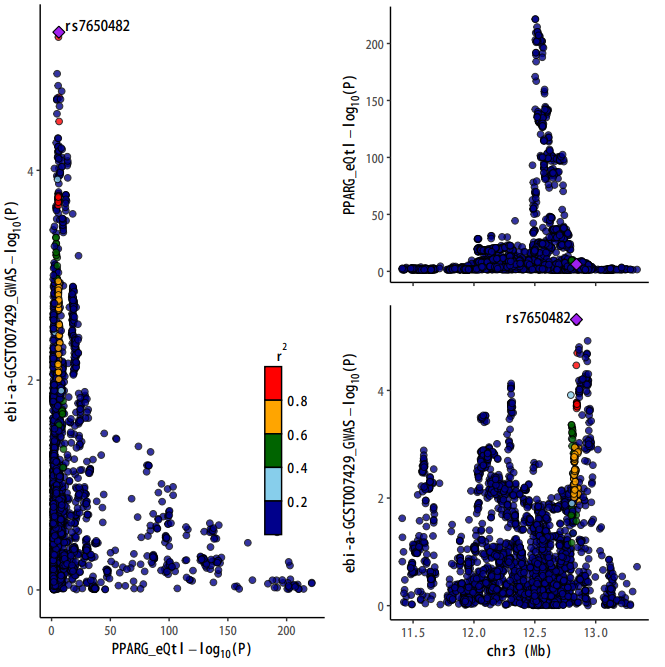


**Figure S13. Results of co-localization analysis of PPARG gene with Forced vital capacity (FVC).**

Each point represents a variant with chromosomal position on the x-axis (within 500-kb regions of each sentinel variant for candidate proteins) and the -log10(p-value) on the y-axis. Variants are coloured by linkage Variants are coloured by linkage disequilibrium with the sentinel variant. Blue lines show the recombination rate; gene locations are shown at the bottom of the plot.


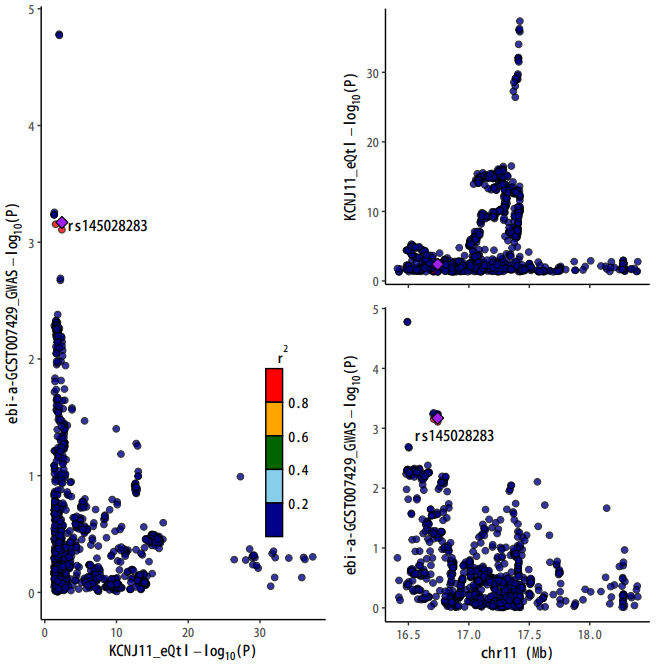


**Figure S14. Results of co-localization analysis of KCNJ11 gene with Forced vital capacity (FVC).**

Each point represents a variant with chromosomal position on the x-axis (within 500-kb regions of each sentinel variant for candidate proteins) and the -log10(p-value) on the y-axis. Variants are coloured by linkage disequilibrium with the sentinel variant. Blue lines show the recombination rate; gene locations are shown at the bottom of the plot.


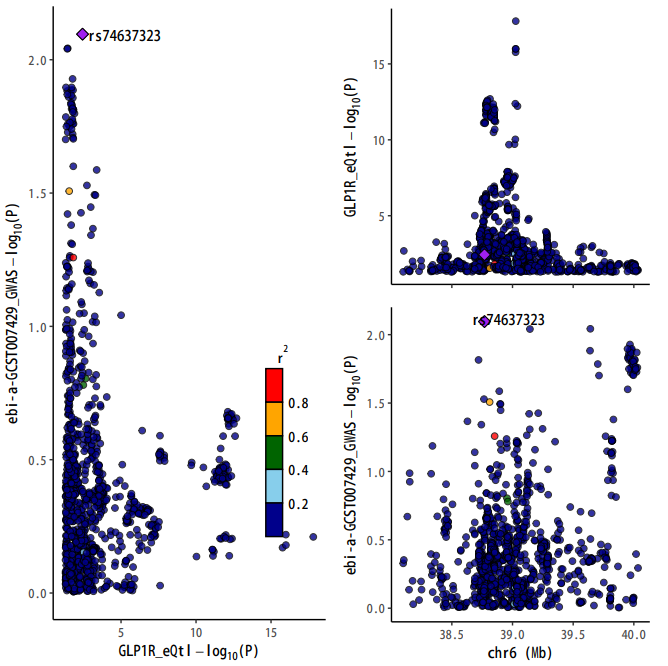


**Figure S15. Results of co-localization analysis of GLP1R gene with Forced vital capacity (FVC).**

Each point represents a variant with chromosomal position on the x-axis (within 500-kb regions of each sentinel variant for candidate proteins) and the -log10(p-value) on the y-axis. Variants are coloured by linkage disequilibrium with the sentinel variant. Blue lines show the recombination rate; gene locations are shown at the bottom of the plot.


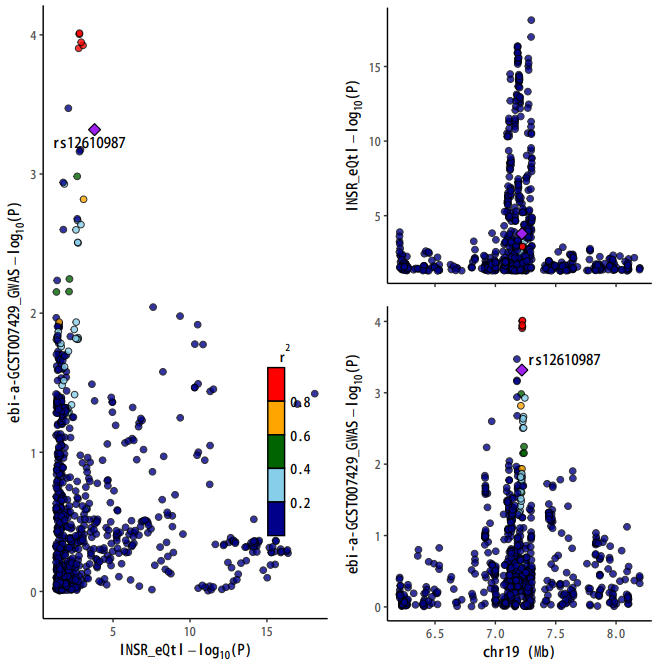


**Figure S16. Results of co-localization analysis of INSR gene with Forced vital capacity (FVC).**

Each point represents a variant with chromosomal position on the x-axis (within 500-kb regions of each sentinel variant for candidate proteins) and the -log10(p-value) on the y-axis. Variants are coloured by linkage disequilibrium with the sentinel variant. Blue lines show the recombination rate; gene locations are shown at the bottom of the plot.


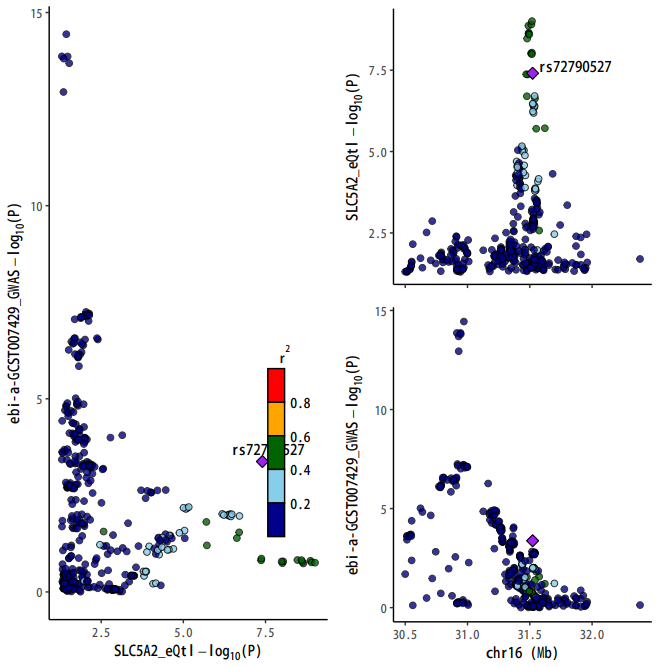


**Figure S17. Results of co-localization analysis of SLC5A2 gene with Forced vital capacity (FVC).**

Each point represents a variant with chromosomal position on the x-axis (within 500-kb regions of each sentinel variant for candidate proteins) and the -log10(p-value) on the y-axis. Variants are coloured by linkage disequilibrium with the sentinel variant. Blue lines show the recombination rate; gene locations are shown at the bottom of the plot.


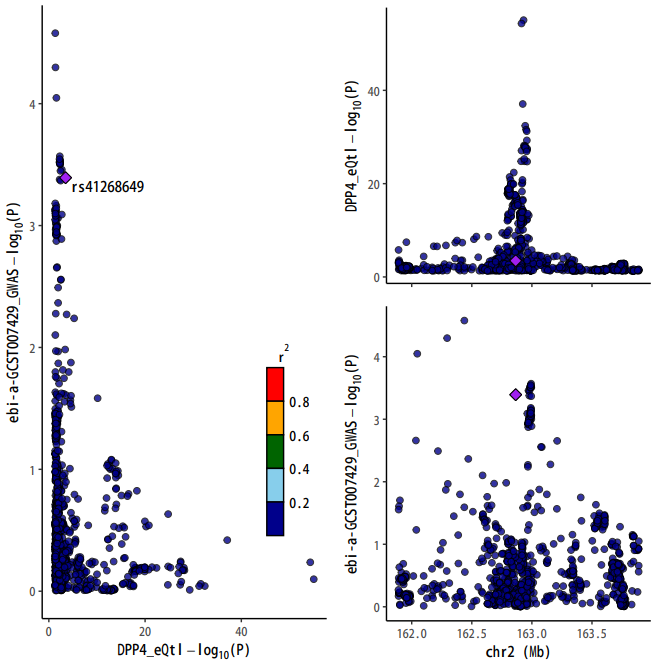


**Figure S18. Results of co-localization analysis of DPP4 gene with Forced vital capacity (FVC).**

Each point represents a variant with chromosomal position on the x-axis (within 500-kb regions of each sentinel variant for candidate proteins) and the -log10(p-value) on the y-axis. Variants are coloured by linkage disequilibrium with the sentinel variant. Blue lines show the recombination rate; gene locations are shown at the bottom of the plot.


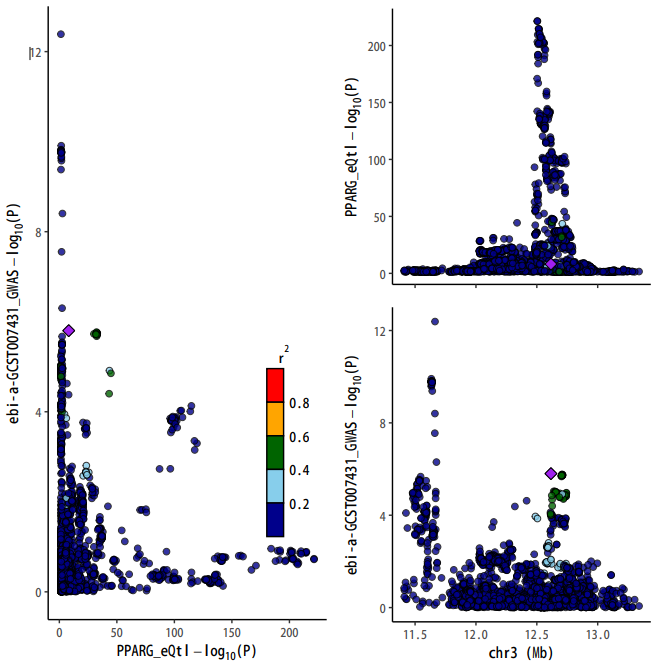


**Figure S19. Results of co-localization analysis of PPARG gene with FEV1/FVC.**

Each point represents a variant with chromosomal position on the x-axis (within 500-kb regions of each sentinel variant for candidate proteins) and the -log10(p-value) on the y-axis. Variants are coloured by linkage disequilibrium with the sentinel variant. Blue lines show the recombination rate; gene locations are shown at the bottom of the plot.


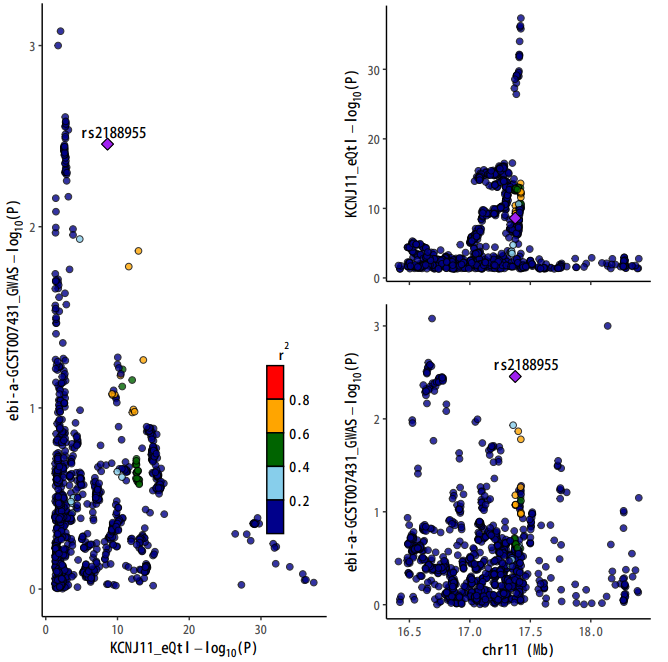


**Figure S20. Results of co-localization analysis of KCNJ11 gene with FEV1/FVC**.

Each point represents a variant with the chromosomal position on the x-axis (within 500-kb regions of each sentinel variant for candidate proteins) and the -log10(p-value) on the y-axis. Variants are coloured by linkage disequilibrium with the sentinel variant. Blue lines show the recombination rate; gene locations are shown at the bottom of the plot.


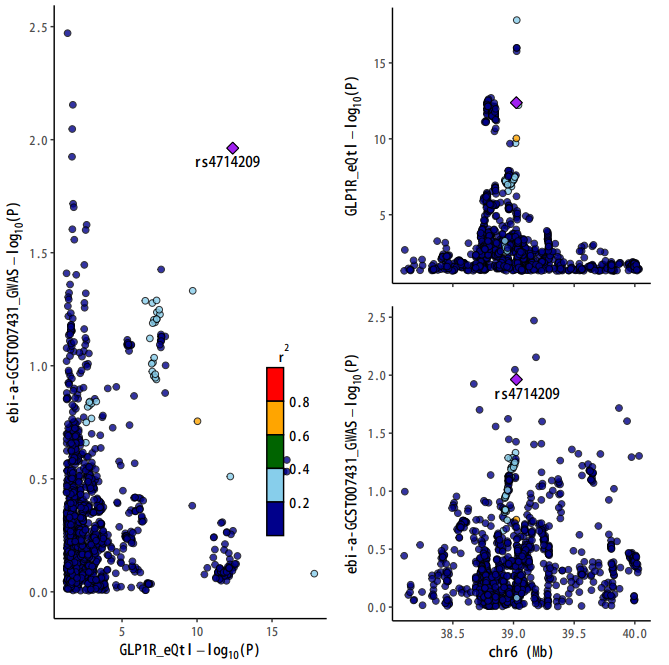


**Figure S21. Results of co-localization analysis of GLP1R gene with FEV1/FVC.**

Each point represents a variant with chromosomal position on the x-axis (within 500-kb regions of each sentinel variant for candidate proteins) and the -log10(p-value) on the y-axis. Variants are coloured by linkage disequilibrium with the sentinel variant. Blue lines show the recombination rate; gene locations are shown at the bottom of the plot.


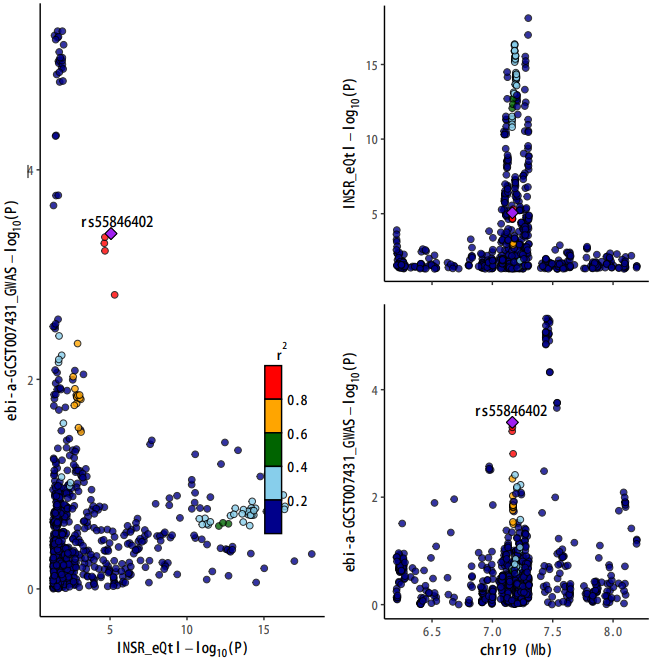


**Figure S22. Results of co-localization analysis of INSR gene with FEV1/FVC.**

Each point represents a variant with chromosomal position on the x-axis (within 500-kb regions of each sentinel variant for candidate proteins) and the -log10(p-value) on the y-axis. Variants are coloured by linkage disequilibrium with the sentinel variant. Blue lines show the recombination rate; gene locations are shown at the bottom of the plot.


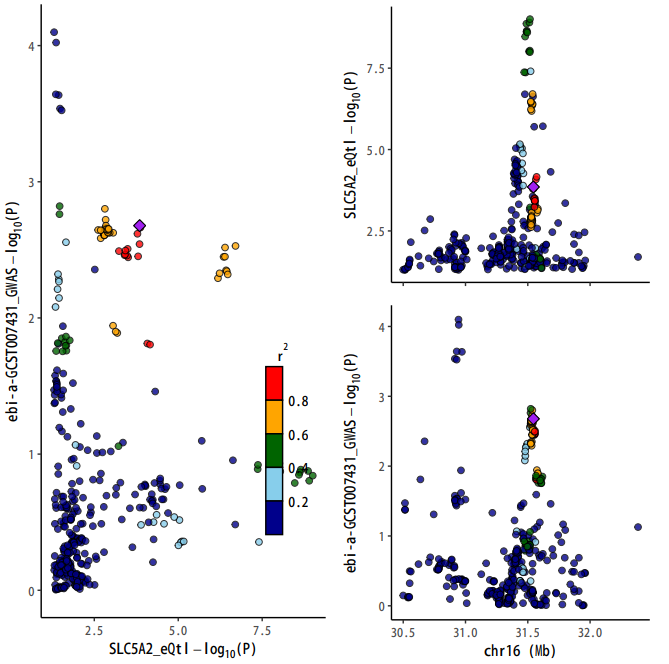


**Figure S23. Results of co-localization analysis of SLC5A2 gene with FEV1/FVC.**

Each point represents a variant with chromosomal position on the x-axis (within 500-kb regions of each sentinel variant for candidate proteins) and the -log10(p-value) on the y-axis. Variants are coloured by linkage disequilibrium with the sentinel variant. Blue lines show the recombination rate; gene locations are shown at the bottom of the plot.


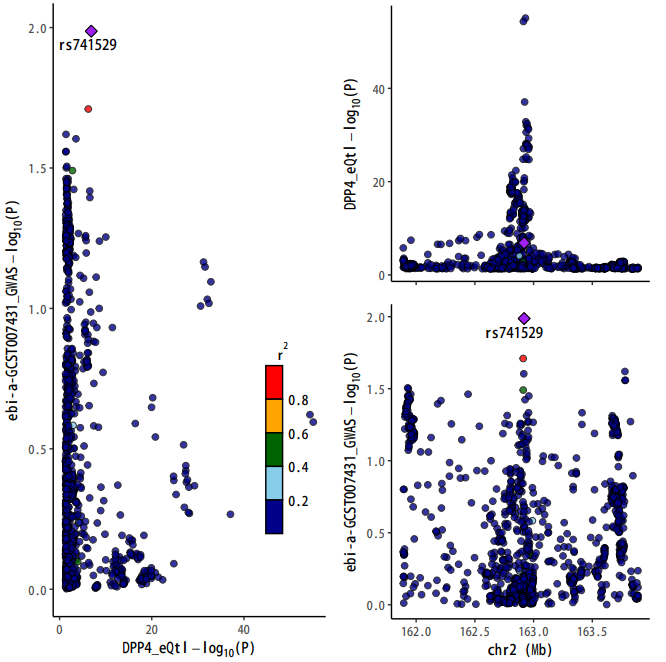


**Figure S24. Results of co-localization analysis of DPP4 gene with FEV1/FVC.**

Each point represents a variant with chromosomal position on the x-axis (within 500-kb regions of each sentinel variant for candidate proteins) and the -log10(p-value) on the y-axis. Variants are coloured by linkage disequilibrium with the sentinel variant. Blue lines show the recombination rate; gene locations are shown at the bottom of the plot.
